# NPC1L1 Deficiency Suppresses Ileal Fibroblast Growth Factor 15 Expression and Increases Bile Acid Pool Size in High-Fat-Diet-Fed Mice

**DOI:** 10.3390/cells10123468

**Published:** 2021-12-09

**Authors:** Lin Jia, Yinyan Ma, Jamie Haywood, Long Jiang, Bingzhong Xue, Hang Shi, Paul A. Dawson, Liqing Yu

**Affiliations:** 1 Department of Pathology, Wake Forest School of Medicine, Winston-Salem, NC 27157, USA; lin.jia@utdallas.edu (L.J.); yinyan.ma@nih.gov (Y.M.); jhaywood@wakehealth.edu (J.H.); paul.dawson@emory.edu (P.A.D.); 2 Department of Biochemistry, Wake Forest School of Medicine, Winston-Salem, NC 27157, USA; 3 Department of Biological Sciences, The University of Texas at Dallas, 800 W, Campbell Road, Richardson, TX 75080, USA; 4 Division of Endocrinology, Diabetes and Nutrition, Department of Medicine, University of Maryland School of Medicine, Baltimore, MD 21201, USA; skyiadx@hotmail.com; 5 Department of Endocrinology and Metabolism, Wake Forest School of Medicine, Winston-Salem, NC 27157, USA; bxue@gsu.edu; 6 Department of Biology, Georgia State University, Atlanta, GA 30303, USA; hshi3@gsu.edu; 7 Internal Medicine Section on Gerontology and Geriatric Medicine, Wake Forest School of Medicine, Winston-Salem, NC 27157, USA; 8 Department of Pediatrics, Division of Gastroenterology, Hepatology, and Nutrition, Emory University, Atlanta, GA 30322, USA

**Keywords:** NPC1L1, cholesterol, obesity, energy expenditure, TGR5

## Abstract

Niemann–Pick C1-like 1 (NPC1L1) mediates intestinal uptake of dietary and biliary cholesterol and is the target of ezetimibe, a cholesterol absorption inhibitor used to treat hypercholesterolemia. Genetic deletion of NPC1L1 or ezetimibe treatment protects mice from high-fat diet (HFD)-induced obesity; however, the molecular mechanisms responsible for this therapeutic benefit remain unknown. A major metabolic fate of cholesterol is its conversion to bile acids. We found that NPC1L1 knockout (L1-KO) mice fed an HFD had increased energy expenditure, bile acid pool size, and fecal bile acid excretion rates. The elevated bile acid pool in the HFD-fed L1-KO mice was enriched with tauro-β-muricholic acid. These changes in the L1-KO mice were associated with reduced ileal mRNA expression of fibroblast growth factor 15 (FGF15) and increased hepatic mRNA expression of cholesterol 7α-hydroxylase (Cyp7A1) and mitochondrial sterol 27-hydroxylase (Cyp27A1). In addition, mRNA expression of the membrane bile acid receptor Takeda G protein-coupled receptor 5 (TGR5) and type 2 iodothyronine deiodinase (Dio2) were elevated in brown adipose tissue of L1-KO mice, which is known to promote energy expenditure. Thus, altered bile acid homeostasis and signaling may play a role in protecting L1-KO mice against HFD-induced obesity.

## 1. Introduction

Niemann–Pick C1-like 1 (NPC1L1) is a polytopic transmembrane protein localized at the brush border membrane of the small intestine [1,2]. It is responsible for intestinal absorption of both biliary and dietary cholesterol [2,3,4,5]. NPC1L1 knockout (L1-KO) mice exhibit greatly reduced intestinal cholesterol absorption and are resistant to high cholesterol-containing diet-induced hypercholesterolemia [2,6]. Ezetimibe is the first of a class of cholesterol-lowering drugs that selectively inhibit cholesterol absorption by targeting NPC1L1 [2,7,8,9]. Surprisingly, in addition to reducing plasma total and low-density lipoprotein (LDL) cholesterol (LDL-C), ezetimibe treatment prevents high-fat diet (HFD)-induced obesity in mice [10] and reduces weight gain in non-obese Japanese males with hypercholesterolemia [11]. Ezetimibe also reduces visceral fat in Japanese patients with metabolic syndrome [12]. Genetic deletion of NPC1L1 protects mice against HFD-induced obesity [13]. Although L1-KO mice do not efficiently absorb intestinal cholesterol [2], the protection against HFD-induced obesity was cholesterol dependent and was reversed when the mice were fed a higher cholesterol-containing HFD [14].

It was reported that L1-KO versus control mice display a 5.2% reduction in intestinal fat absorption [13]. The authors estimated that this reduction in fat absorption may not account for all of the differences in weight gain between the two genotypes [13]. We observed a 7.8% reduction in intestinal fat absorption in L1-KO mice fed a *trans*-fat (hydrogenated vegetable oil)-based HFD [15] but not in those fed a lard-based HFD [14]. However, the L1-KO mice fed either diet gained less weight versus control mice. In addition, we and others did not detect any significant differences in food or energy intake between L1-KO mice or ezetimibe-treated mice and their respective controls [10,13,14]. These observations collectively suggest that mechanisms in addition to intestinal fat absorption may be involved in protecting L1-KO mice from HFD-induced weight gain.

Bile acids are synthesized from cholesterol in the liver, stored in the gallbladder, and released postprandially into the lumen of the small intestine where they facilitate digestion and absorption of dietary fat, cholesterol, and lipid-soluble vitamins. More than 90% of bile acids are reabsorbed by enterocytes in the ileum via the apically localized bile acid transporter, the apical sodium-dependent bile acid transporter (ASBT), and the basolaterally localized bile acid efflux transporter, the organic solute transporter (Ost)α/Ostβ heterodimer [16,17]. The reabsorbed bile acids are returned to the liver via the portal vein. This enterohepatic recirculation of bile acids is important for nutrient absorption and bile acid homeostasis. Bile acid biosynthesis is subject to feedback regulation. Increases in bile acids activate the nuclear hormone receptor, farnesoid X receptor (FXR) [18,19]. In the liver, FXR activation inhibits cholesterol 7α hydroxylase (Cyp7A1), the rate-limiting enzyme of bile acid biosynthesis, to repress bile acid synthesis [18,19]. In the distal small intestine, FXR activation increases fibroblast growth factor 15 (FGF15; in humans, FGF19) expression and secretion [20]. Secreted FGF15 travels to the liver via the portal vein. In the liver, FGF15 activates its receptor FGF receptor 4 (FGFR4) and signals to suppress Cyp7A1 transcription and bile acid synthesis [20]. Under physiological conditions, this is the major pathway for negative feedback regulation of hepatic bile acid synthesis.

In addition to the nuclear receptor FXR, G protein-coupled bile acid receptor 1 (GPBAR1, also known as Takeda G protein-coupled receptor 5 or TGR5) was identified as a membrane-type receptor for bile acids [21,22]. Activation of TGR5 by bile acids raises intracellular cyclic AMP (cAMP), which then increases transcription and activity of type 2 iodothyronine deiodinase (Dio2), an enzyme that converts inactive T4 to active T3 locally in the brown adipose tissue (BAT) [23]. The increased levels of T3 in BAT were shown to mediate the bile acid feeding-induced increase in energy expenditure and prevent diet-induced obesity in mice [23].

NPC1L1 deficiency dramatically alters whole-body cholesterol homeostasis [2,3,4,5]. Since cholesterol is the precursor for bile acid biosynthesis, we hypothesized that NPC1L1 deficiency may alter bile acid homeostasis. Considering the role of the bile acid-TGR5-Dio2 signaling in thermogenesis [23], an energy-dissipating process, we further hypothesized that altered bile acid homeostasis may protect L1-KO mice from HFD-induced obesity by increasing energy expenditure through the TGR5-Dio2 pathway. Consistent with our hypotheses, we observed that L1-KO mice, compared with their littermate wild-type (WT) controls fed an HFD, had a significant increase in energy expenditure, bile acid pool size, and enrichment of tauro-β-muricholate (TBMC) in the bile acid pool. These animals also had reduced ileal expression of FGF15, increased hepatic expression of Cyp7A1 and Cyp27A1, and elevated mRNAs levels of TGR5 and Dio2 in BAT.

## 2. Materials and Methods

### 2.1. Animals and Diets

L1-KO mice were created by using embryonic stem cells from pure C57BL/6J mice and the standard gene-targeting approach [6] (kindly provided by Drs. Yannis Ioannou and Joanna P. Davies at Mount Sinai School of Medicine in New York). All mice were housed in a specific pathogen-free animal facility in plastic cages at 22 °C, with a daylight cycle from 6 a.m. to 6 p.m. The mice were provided with water and a standard chow diet ad libitum unless stated otherwise. All animal procedures were approved by the Institutional Animal Care and Use Committees at Wake Forest University Health Sciences and the University of Maryland School of Medicine. Male L1-KO mice and their WT controls were fed an HFD (TD.93075; Envigo, Madison, WI, USA) for 6 or 24 weeks, starting at 6 weeks of age. The HFD derives 54.8% calories from fat, 21.2% calories from protein, and 24% calories from carbohydrates. It contains only a trace amount of cholesterol (~0.007%). The fatty acid composition in the fat of this diet is 28% saturated, 30% monounsaturated-(*trans*), 28% monounsaturated-(*cis*), and 14% polyunsaturated-(*cis*) fatty acids.

### 2.2. Indirect Calorimetry

Indirect calorimetry was performed in the Mouse Metabolic Phenotyping Center at Vanderbilt University Medical Center by using an Oxymax indirect calorimeter (Columbus Instruments, Columbus, OH, USA) with an airflow of 0.75 L/min. Oxygen consumption, carbon dioxide production, and energy expenditure were normalized using the metabolic body size [kilogram (kg)^0.75^ body weight (BW)] as described by others [23,24].

### 2.3. Measurements of Biliary Cholesterol, Bile Acids, and Phospholipids

A measured volume (5–10 µL) of gallbladder bile was placed into a glass tube. Biliary lipids were extracted by the Bligh–Dyer method [25] in the presence of 10 μg 5α-cholestane as an internal standard. The upper aqueous phase was analyzed for the total bile acid content using an enzymatic assay as previously described [26]. The bottom organic phase was analyzed for free cholesterol content by gas–liquid chromatography and for phospholipid (PL) content using Phospholipids B enzymatic assay kit (Wako).

### 2.4. Measurements of Plasma Bile Acid Concentrations, Fecal Bile Acid Excretion, and Bile Acid Pool Size

Plasma was collected after a 4 h fast, and concentrations of bile acids were measured at the Emory Integrated Lipidomics Core by UPLC–MS/MS with an Infinity 1295 II UPLC, Zorbax Eclipse Plus C18 column, and Agilent 6495C mass spectrometer using a dynamic MRM method for targeted analysis and standards for all the major mouse bile acid species. Fecal bile acid excretion was measured in the mice fed the HFD for 18 weeks. Feces were collected from these HFD-fed mice that had been housed individually in the wire-bottom cages for 72 h and extracted as described previously [27]. The total bile acid content in the fecal extract was determined enzymatically [28]. The bile acid pool size was analyzed in the mice fed the HFD for 24 weeks as described [29]. After a 4 h fast, the small intestine plus luminal contents, gallbladder, and liver were collected. Bile acids were extracted and the bile acid composition was determined using the high-performance liquid chromatography (HPLC) [29,30,31]. Individual bile acid species were detected by using an evaporative light scattering detector (Alltech ELSD 800) and quantified by comparison with authentic standards purchased from Steraloids.

### 2.5. Quantitative Real-Time PCR (qPCR)

Measurements of the mRNA levels for selected genes in the ileum, liver, and interscapular BAT were performed as described previously [32]. Primers sequences are listed as follows: FXR, forward, TGAGAACCCACAGCATTTCG, and reverse, GCGTGGTGATGGTTGAATGTC; ASBT, forward, TGGGTTTCTTCCTGGCTAGACT, and reverse, TGTTCTGCATTCCAGTTTCCAA; Ostα, forward, TACAAGAACACCCTTTGCCC, and reverse, CGAGGAATCCAGAGACCAAA; Ostβ, forward, GTATTTTCGTGCAGAAGATGCG, and reverse, TTTCTGTTTGCCAGGATGCTC; ILBP, forward, CAAGGCTACCGTGAAGATGGA, and reverse, CCCACGACCTCCGAAGTCT; FGF15, forward, GCTCTGAAGACGATTGCCATC, and reverse, TTCCTCCCTGAAGGTACAGTC; Cyp7A1, forward, AGCAACTAAACAACCTGCCAGTACTA, and reverse, GTCCGGATATTCAAGGATGCA; Cyp27A1, forward, GGAGGGCAAGTACCCAATAAGA, and reverse, TGCGATGAAGATCCCATAGGT; Cyp8B1, forward, GCCTTCAAGTATGATCGGTTCCT, and reverse, GATCTTCTTGCCCGACTTGTAGA; SHP, forward, CAGCGCTGCCTGGAGTCT, and reverse, AGGATCGTGCCCTTCAGGTA; HNF4, forward, ACTGTCCAGAGCTAGCGGAGAT, and reverse, GCAGGCATATTCATTGTCATCAA; TGR5, forward, TGGGTCAGCTCCCTGTTCTT, and reverse, TGGCATCAGGGCTCCAAT; Dio2, forward, CAGCTTCCTCCTAGATGCCTACA, and reverse, GACGTGCACCACACTGGAAT; UCP1, forward, GAGGTGTGGCAGTGTTCATTG, and reverse, GGCTTGCATTCTGACCTTCA; PGC1α, forward, AACCACACCCACAGGATCAGA, and reverse, TCTTCGCTTTATTGCTCCATGA; PPARα, forward, ACAAGGCCTCAGGGTACCA, and reverse, GCCGAAAGAAGCCCTTACAG; PPARγ, forward, CACAATGCCATCAGGTTTGG, and reverse, GCTGGTCGATATCACTGGAGATC; CTP1A, forward, CACCAACGGGCTCATCTTCTA, and reverse, CAAAATGACCTAGCCTTCTATCGAA.

### 2.6. Statistical Analysis

Data are expressed as mean ± standard error of the mean (SEM). The difference between the mean values of L1-KO and WT groups was tested for statistical significance by two-tailed Student’s *t*-tests. A value of *p* < 0.05 was accepted as statistically significant.

## 3. Results

### 3.1. NPC1L1 Deficiency in Mice Increases Energy Expenditure

**Figure 1:**
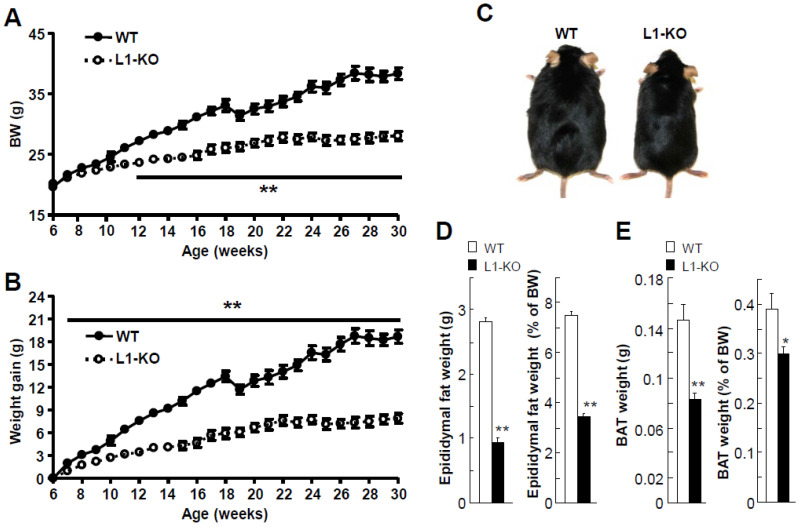
Reduced weight gain and fat tissue weights in L1-KO male mice fed the low-cholesterol (0.007%, *w*/*w*)-containing HFD: (**A**,**B**) body weight (BW) curves (**A**) and weight gain (**B**) during HFD feeding; (**C**–**E**) gross appearance of L1-KO mouse and WT littermate (**C**), epididymal fat pad weight and its ratio to BW (**D**), and interscapular BAT weight and its ratio to BW (**E**) after 24 weeks of HFD feeding. * *p* < 0.05, ** *p* < 0.001 (*n* = 6 in WT group vs. *n* = 8 in L1-KO group). The weight drop occurred during the transfer of mice from one building to the other.

We and others have shown that NPC1L1 inhibition or deletion protects mice against HFD-induced obesity [10,13,14]. However, it was unknown whether this protection was associated with increased energy expenditure or altered bile acid homeostasis. To address these questions, in this study, we fed L1-KO and their WT controls a low-cholesterol-containing HFD and analyzed energy and bile acid metabolism in these animals. We had previously shown that the weight gain differences between L1-KO and control mice were observed when the mice were fed an HFD without added cholesterol [14]. As expected, L1-KO mice weighed significantly less than WT mice after 6 weeks on the HFD (Figure 1A). Although both genotypes had similar body weights before HFD feeding, reduced weight gain in L1-KO mice was observed as early as one week on the HFD (Figure 1B). The weight loss of WT mice in the 19th week of age resulted from animal transport to a different building, highlighting an important effect of the housing environment on animal health. After 24 weeks of HFD challenge, L1-KO mice, compared with WT controls, appeared leaner (Figure 1C). The reduced weight gain of L1-KO mice was associated with a decrease in epididymal and brown fat weights (Figure 1D,E).

**Figure 2:**
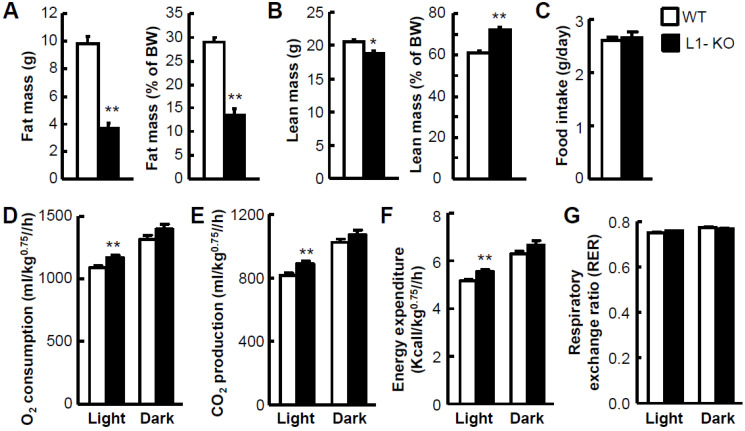
Reduced whole-body adiposity and increased energy expenditure in L1-KO mice fed the HFD for 23–25 weeks: (**A**,**B**) fat body mass and its ratio to BW (**A**) and lean body mass and its ratio to BW (**B**) in L1-KO mice (*n* = 10) and WT littermates (*n* = 9); (**C**–**G**) food intake (**C**), oxygen (O2) consumption (**D**), carbon dioxide (CO2) production (**E**), energy expenditure (**F**), and respiratory exchange ratio (RER) (**G**) in L1-KO mice (*n* = 15) and WT littermates (*n* = 12). * *p* < 0.01, ** *p* < 0.000001.

To determine whether the reduced weight gain of L1-KO mice was a result of reduced fat mass but not lean mass in the whole body, the body composition of L1-KO and WT mice was analyzed by NMR at Vanderbilt’s Mouse Metabolic Phenotyping Center. L1-KO mice displayed a 60% decrease in the fat body mass (Figure 2A) but only an 8.8% reduction in the lean body mass (Figure 2B). When normalized to BW, the fat mass-to-BW ratio decreased 53.2%, while the lean mass-to-BW ratio increased 17.8% in L1-KO mice, compared with control mice (Figure 2A,B). The reduced weight gain and fat mass in L1-KO mice were not a result of reduced food intake (Figure 2C). The physical activities were not different either between the two groups (data not shown). When whole-body energy expenditure was measured using the Indirect Calorimetry, L1-KO mice versus WT mice had significantly increased oxygen (O_2_) consumption (Figure 2D), carbon dioxide (CO_2_) production (Figure 2E), and energy expenditure (Figure 2F) in the light cycle, though no significant changes in the dark cycle. The respiratory exchange ratio (RER) was comparable between the two genotypes (Figure 2G), suggesting that NPC1L1 deficiency did not alter the relative contribution of fat and carbohydrates to energy metabolism under the current experimental conditions.

### 3.2. NPC1L1 Deficiency in Mice Increases Biliary Bile Acids and Cholesterol

**Figure 3:**
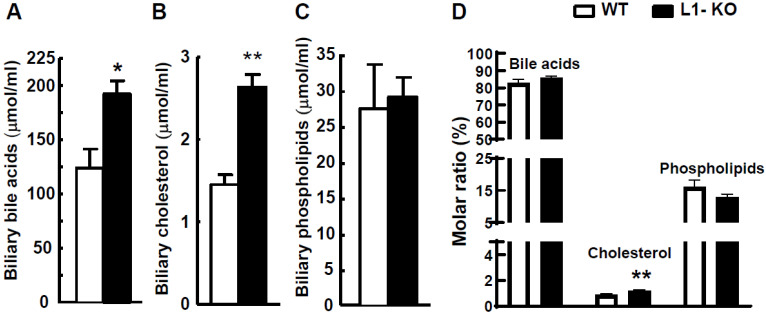
L1-KO mice have increased biliary concentrations of bile acids and cholesterol: (**A**–**C**) biliary concentrations of bile acids (**A**), cholesterol (**B**), and phospholipids (**C**) in the gallbladder bile of L1-KO and WT mice fed the HFD for 24 weeks; (**D**) molar ratios of biliary bile acids, cholesterol, and phospholipids calculated from the data shown in (**A**–**C**). * *p* < 0.05, ** *p* < 0.01 (*n* = 6–8).

Cholesterol homeostasis is substantially altered in L1-KO mice [2,3,33]. Bile acids are end products of cholesterol catabolism and play important roles in regulating energy expenditure in mice [23,34]. To determine whether NPC1L1-deficient mice fed the HFD have altered bile acid metabolism, we first examined biliary concentrations of bile acids and two major lipids (cholesterol and phospholipids) in the gallbladder. After 24 weeks of HFD feeding, L1-KO, compared with WT mice, showed a 55% increase in biliary bile acids (Figure 3A), an 81% increase in biliary cholesterol (Figure 3B), and no alterations in biliary phospholipids (Figure 3C). The calculation of the molar ratio (Figure 3D) showed that HFD-fed L1-KO mice had significantly elevated molar percent of biliary cholesterol. In addition, the molar percent of biliary bile acid was slightly increased in L1-KO mice after long-term HFD feeding.

### 3.3. NPC1L1 Deficiency in Mice Increases Bile Acid Pool Size

**Figure 4:**
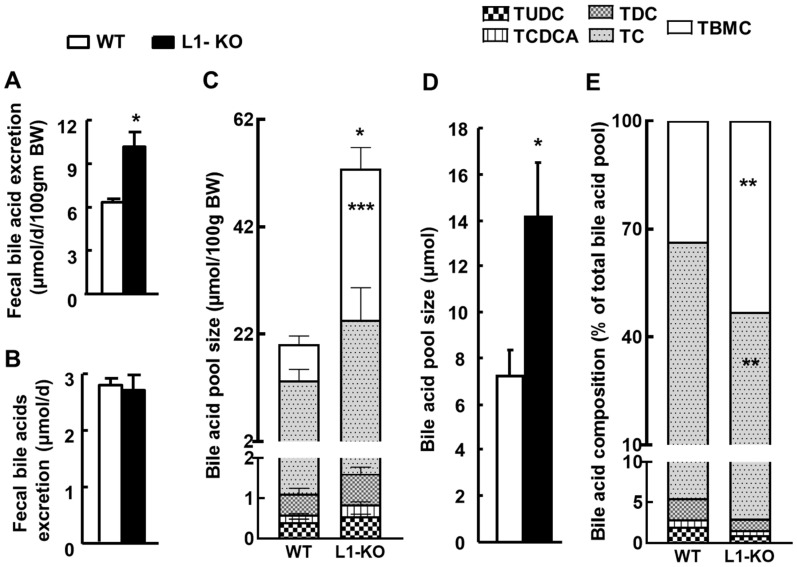
L1-KO mice have increased bile acid pool size and elevated TBMC in the bile acid pool after 18 weeks on the HFD: (**A**) fecal bile acid excretion normalized by BW; (**B**) fecal bile acid excretion without BW normalization; (**C**) bile acid pool size; (**D**) bile acid pool size without BW normalization; (**E**) bile acid composition. * *p* < 0.05, ** *p* < 0.01, *** *p* < 0.001 (*n* = 6–8). TUDC, tauroursodeoxycholate; TCDCA, taurochenodeoxycholic acid; TDC, taurodeoxycholate; TC, taurocholate; TBMC, tauro-β-muricholate.

Next, we examined whether whole-body bile acid homeostasis was changed in L1-KO mice. After 24 weeks on the HFD, L1-KO mice, compared with WT mice, exhibited a significant increase in fecal bile acid excretion rate (Figure 4A), and this increase was attributable to the lower BW of L1-KO mice. Without normalization to BW, the daily fecal bile acid output was similar between the two genotypes of mice (Figure 4B). In addition, L1-KO mice had a significant increase in the bile acid pool size, with the largest increase seen in the TBMC pool (Figure 4C), which increased from 6.7 ± 1.6 μmol/100 g BW in WT mice to 28.2 ± 3.9 μmol/100 g BW in L1-KO mice (*p* < 0.001). The increase in the bile acid pool size was independent of BW because it remained significantly increased without normalization to BW (Figure 4D). When bile acid compositions in the pool were calculated, the relative content of TBMC was significantly enriched, whereas that of taurocholate (TC) was significantly reduced in L1-KO mice, compared with WT controls (Figure 4E).

### 3.4. NPC1L1 Deficiency in Mice Reduces Ileal Expression of FGF15

**Figure 5:**
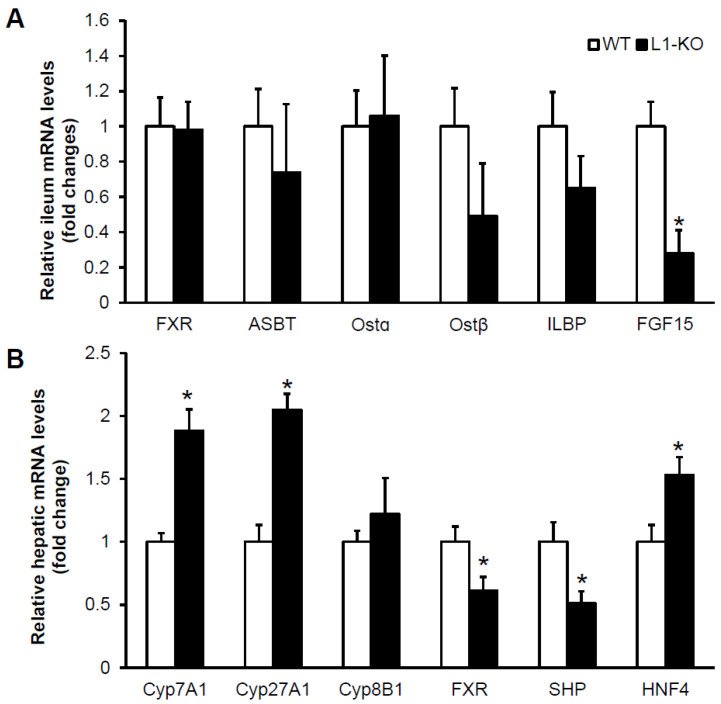
L1-KO mice have reduced ileal expression of FGF15 mRNA and increased hepatic expression of mRNAs for Cyp7A1 and Cyp27A1 after 6 weeks of HFD feeding: (**A**,**B**) relative mRNA levels in the distal ileum (**A**) and liver (**B**) of L1-KO mice and WT littermates measured by qPCR using individual total RNA samples in each group (*n* = 5). GAPDH and 18S RNAs were used as internal invariant controls for the ileum and liver, respectively. * *p* < 0.05.

To probe potential mechanisms underlying altered bile acid homeostasis in the HFD-fed L1-KO mice, we measured ileal and hepatic mRNAs for genes involved in the metabolism and regulation of bile acids in L1-KO and control mice fed the HFD for 6 weeks. This dietary time point was chosen because it was when the two genotypes of mice began to show BW differences (Figure 1A), and we wanted to minimize potential secondary effects of large BW differences in gene expression after long-term HFD feeding. We found that ileal levels of mRNAs for FXR, ASBT, Ostα, Ostβ, and ileal lipid-binding protein (ILBP) were similar between the two genotypes (Figure 5A). Interestingly, FGF15 mRNA expression significantly decreased 72% in the ileum of L1-KO mice (Figure 5A). Decreased intestinal FGF15 is predicted to upregulate hepatic Cyp7A1 expression [20], the rate-limiting enzyme in bile acid biosynthesis. Indeed, the hepatic Cyp7A1 mRNA level increased 1.9-fold in L1-KO mice (Figure 5B). Additionally, the hepatic mRNA for Cyp27A1, the initiating enzyme in the alternative (acidic) pathway of bile acid synthesis [35], elevated 2.1-fold in the HFD-fed L1-KO mice, compared with control mice (Figure 5B). No genotypic differences were observed in hepatic levels of mRNA for sterol 12α-hydroxylase (Cyp8B1), an enzyme that is required for cholic acid synthesis [36] (Figure 5B). These changes in hepatic mRNA levels of bile acid synthetic enzymes in the L1-KO mice were associated with reduced mRNAs for FXR and SHP and increased mRNA for hepatocyte nuclear factor 4 (HNF4) in the liver (Figure 5B).

### 3.5. NPC1L1 Deficiency Increases TGR5 and Dio2 Expression in BAT

**Figure 6:**
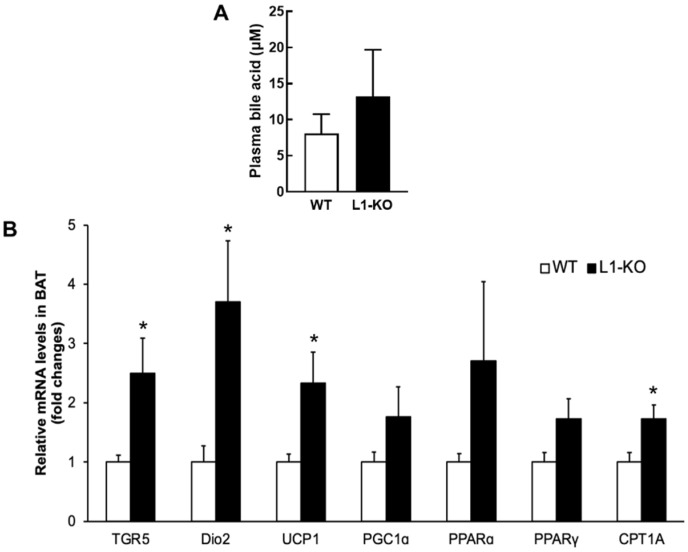
L1-KO mice have increased mRNA levels of genes involved in adaptive thermogenesis and fatty acid oxidation in BAT: (**A**) plasma concentrations of bile acids analyzed by UPLC–MS/MS in WT and L1-KO mice after 6 weeks of HDF feeding (*n* = 8); (**B**) relative mRNA levels in the interscapular BAT measured by qPCR with individual total RNA samples in each group (*n* = 5). For this study, 18S RNA was used as an internal invariant control. * *p* < 0.05.

It has been shown that bile acids promote energy expenditure by activating the TGR5-Dio2 pathway in BAT [23]. To determine whether altered bile acid homeostasis in the HFD-fed L1-KO mice was associated with any changes in this pathway, we measured circulating bile acid concentrations and the mRNA expression levels of genes related to energy expenditure in the interscapular BAT of L1-KO and control mice fed the HFD for 6 weeks. We found that L1-KO mice exhibited an elevated plasma total bile acid concentration (Figure 6A). In addition, a significant increase was observed in BAT levels of mRNAs for TGR5, Dio2, uncoupling protein 1 (UCP-1), and carnitine palmitoyltransferase 1A (CPT1A) in L1-KO mice, compared with the controls (Figure 6B). BAT mRNA levels of peroxisome proliferator-activated receptor (PPAR) gamma coactivator-1α (PGC1α), PPARα, and PPARγ showed a trend towards an increase in the L1-KO mice, but the increase did not reach statistical significance (Figure 6B).

## 4. Discussion

NPC1L1 is essential for intestinal absorption of cholesterol in the gut lumen [2]. NPC1L1 deficiency protects mice against obesity induced by various HFDs that are low in cholesterol [13,14]. It is currently unknown how NPC1L1 modulates diet-induced obesity. In this study, we demonstrate that L1-KO versus WT mice fed an HFD display increased energy expenditure and bile acid pool size. These changes are associated with reduced ileal mRNA levels of FGF15 and increased BAT mRNA levels of TGR5 and Dio2. It has been shown that activation of the TGR5-Dio2 pathway by bile acids promotes energy expenditure in mice [23]. Future studies are warranted to directly test whether BAT TGR5 or Dio2 is required for L1-KO mice to resist HFD-induced obesity. With regard to the effects on bile acid pool size, a significant reduction in ileal FGF15 expression was observed in L1-KO mice in the present study (Figure 5A), in which mice were fed a low-cholesterol-containing HFD (TD.93075; Envigo), which contains ~30% monounsaturated-(*trans*) fat. In addition, a similar decrease in ileal FGF15 mRNA expression was also observed when L1-KO mice were fed a different low-cholesterol-containing HFD (D12492; Research Diets), which has no *trans* fat (data not shown). We also observed previously that mice treated with the NPC1L1 inhibitor ezetimibe express significantly lower levels of FGF15 in the small intestine [37]. Intestine-released FGF15 is known to inhibit bile acid synthesis in the liver by suppressing transcription of Cyp7A1 through the cooperation of FGFR4 and SHP [20]. Therefore, the reduced expression of intestinal FGF15 in L1-KO mice may alleviate suppression of Cyp7A1 transcription in the liver, thereby increasing bile acid synthesis and pool size. In agreement with this hypothesis, HFD-fed L1-KO mice exhibited increased hepatic mRNA expression of Cyp7A1 and Cyp27A1, and fecal bile acid excretion (Figure 4A). The increase in hepatic Cyp7a1 and Cyp27a1 mRNA expression was associated with reduced FXR and SHP expression and increased HNF4 expression. This expression pattern of FXR, SHP, and HNF4 transcriptional factors in the liver is consistent with their reported roles in the regulation of hepatic Cyp7A1 and Cyp27A1 expression and bile acid synthesis [20,38,39,40,41,42].

FGF15 and SHP have been shown to suppress NPC1L1 expression through inhibition of sterol regulatory element-binding transcription factor 2 (SREBP2) in the mouse intestine [43]. SREBP2-mediated endogenous cholesterol synthesis is essential for sustaining the health of intestinal mucosa when NPC1L1 is inhibited [44]. Together with our finding that NPC1L1 deficiency suppresses intestinal FGF15 expression (Figure 5A), there seems to exist a reciprocal regulation of FGF15 and NPC1L1 in the mouse intestine. It would be interesting to determine whether the SREBP2 and SHP interaction is implicated in this regulation. Alternatively, the reduced ileal FGF15 expression may be secondary to altered cholesterol and bile acid homeostasis. The reduced ileal FGF15 may result from changes in the bile acid compositions in the pool that is enriched with TBMC. TBMC has been identified as a FXR antagonist [45,46]. While increased hepatic expression of Cyp7A1 and Cyp27A1 likely accounts for bile acid pool size expansion, the mechanism underlying TBMC enrichment is unclear. Cyp8B1 deficiency is known to shift bile acid pool compositions from cholic acid to muricholic acid [36]. Overexpression of Cyp8B1 reduces chenodeoxycholic acid (CDCA) and its muricholic acid derivatives in the bile acid pool [47]. We did not detect significant changes in hepatic Cyp8B1 mRNA levels in the L1-KO mice fed the HFD for 6 weeks. It has been reported that germ-free mice, compared with the conventionally raised mice, display increased bile acid pool size, enriched TBMC in the pool, reduced FXR signaling, and increased bile acid synthesis [45], which mirror our findings in the HFD-fed L1-KO mice. Germ-free mice also express reduced FGF15 mRNA levels in the small intestine [37,45]. L1-KO mice or mice treated with the NPC1L1 inhibitor ezetimibe show reduced stool output, despite similar food intake [14,37,48]. Reduced stool output was also seen in germ-free mice [37], suggesting a significant contribution of gut bacteria to the stool output. Since NPC1L1 deficiency blocks intestinal cholesterol absorption, the amount of cholesterol passing through the gut lumen and in the feces is significantly increased [2,49], which has the potential to shape the gut microbiome. Indeed, L1-KO versus control mice display substantial alterations in the composition of gut microbiota [37]. Perhaps, NPC1L1 deficiency increases bile acid synthesis and pool size and shifts the bile acid composition toward TBMC by modifying the gut microbiome.

Bile acid treatment has been shown to increase energy expenditure by activating the TGR5-Dio2 signaling in BAT and skeletal muscle [23]. Subjects after gastric bypass surgery lose weight and have increased serum bile acid concentrations [50]. In this study, L1-KO mice exhibit increased expression of TGR5 and Dio2 and other thermogenic/oxidative genes, which was associated with a 61% increase in total plasma bile acids (Figure 6A). Interestingly, not all bile acid species interact with TGR5 equally and the bile acid composition may be as important as the total concentration [21,22]. Although we observed slightly increased plasma bile acid concentrations in L1-KO mice, we cannot rule out the possibility that certain species of bile acid could be elevated and activate TGR5 in the brown adipose tissue. Additionally, the relationship between circulating bile acids and metabolic activity appears to be complicated [51,52]. There was a report that colestimide, a bile-acid-binding resin that reduces plasma bile acids [53], prevents mice from HFD-induced obesity and insulin resistance [34]. Alternatively, bile acids can promote energy expenditure by stimulating glucagon-like peptide 1 (GLP-1) secretion from intestinal endocrine cells after binding to TGR5 in these specialized cells [54,55]. However, we did not observe elevated plasma GLP-1 levels in the HFD-fed L1-KO mice (data not shown).

We and others have reported that L1-KO or ezetimibe-treated mice show dramatically reduced liver cholesterol contents [3,14,15]. In addition, we have shown that HFD-fed L1-KO mice exhibited significantly increased (approximately sevenfold) hepatic endogenous cholesterol synthesis [14]. This elevated de novo cholesterol synthesis in the liver could contribute to the increased biliary cholesterol content observed in L1-KO mice after low-cholesterol-containing HFD feeding (Figure 3B). Consistently, ezetimibe treatment increases biliary cholesterol levels when mice were fed a diet containing a low amount of cholesterol [56]. In addition, ezetimibe-treated hamsters on a basal diet without added cholesterol also show increased biliary cholesterol concentrations [57]. Importantly, this study also reported similar hepatic ATP-binding cassette transporters ABCG5 (G5) and ABCG8 (G8) (ABCG5/G8) expression levels in hamsters regardless of the treatment [57]. We also observed previously that L1-KO mice or NPC1L1 inhibitor ezetimibe-treated mice, compared with their controls, express similar levels of ABCG5 protein in the liver [37,48]. Therefore, it is unlikely that the increased biliary cholesterol is a result of increased ABCG5/G8 expression. While increased cholesterol synthesis may passively leak some cholesterol into the bile of L1-KO mice, we cannot exclude a role of gallbladder NPC1L1 deficiency. We have previously shown that NPC1L1 is abundantly expressed in the epithelium of the mouse gallbladder [58]. NPC1L1 protein in this location may mediate reabsorption of free cholesterol from the gallbladder and its deficiency may lead to an increase in gallbladder cholesterol. Regarding the tissue distribution of NPC1L1 expression, in rodents, NPC1L1 is almost exclusively expressed in the small intestine and gallbladder. However, humans and non-human primates express a significant amount of NPC1L1 in the liver as well. Thus, the potential contribution of altered hepatic NPC1L1 expression to increased biliary cholesterol content, if any, should be limited in mice.

In conclusion, NPC1L1-deficient mice fed a low-cholesterol-containing HFD have increased bile acid pool size, enriched TBMC in the bile acid pool, and augmented hepatic expression of Cyp7A1 and Cyp27A1, which is linked to reduced expression of ileal FGF15. Altered bile acid homeostasis may account, in part, for increased energy expenditure and associated lean phenotype via the TGR5-Dio2 pathway in these animals.
